# Leaky-Wave Radiations by Modulating Surface Impedance on Subwavelength Corrugated Metal Structures

**DOI:** 10.1038/srep23974

**Published:** 2016-04-01

**Authors:** Ben Geng Cai, Yun Bo Li, Hui Feng Ma, Wei Xiang Jiang, Qiang Cheng, Tie Jun Cui

**Affiliations:** 1 State Key Laboratory of Millimeter Waves, Department of Radio Engineering Southeast University, Nanjing 210096, China; 2 Synergetic Innovation Center of Wireless Communication Technology, Southeast University, Nanjing, 210096, China; 3 Cooperative Innovation Centre of Terahertz Science, No. 4, Section 2, North Jianshe Road, Chengdu 610054, China

## Abstract

One-dimensional (1D) subwavelength corrugated metal structures has been described to support spoof surface plasmon polaritons (SPPs). Here we demonstrate that a periodically modulated 1D subwavelength corrugated metal structure can convert spoof SPPs to propagating waves. The structure is fed at the center through a slit with a connected waveguide on the input side. The subwavelength corrugated metal structure on the output surface is regarded as metasurface and modulated periodically to realize the leaky-wave radiation at the broadside. The surface impedance of the corrugated metal structure is modulated by using cosine function and triangle-wave function, respectively, to reach the radiation effect. Full wave simulations and measuremental results are presented to validate the proposed design.

After the phenomenon of extraordinary transmissions through subwavelength holes was investigated by surface plasmon polaritons (SPPs) between metal and air in the optical region^1^,^2^, it was found that the corrugated metal structure on the output surface could shape the transmitted light emerging from the aperture^2^,^3^,^4^. The field on the exit surface has also been analyzed using the leaky surface-plasmon theory^5^,^6^,^7^,^8^ and transforamtion optics^9^. In the microwave region, a two-dimensional (2D) metal with drilling holes was proposed and investigated to support spoof SPPs^10^. The one-dimensional (1D) corrugated metal structure has also shown to propagate spoof SPPs^11^. Similarly, electromagnetic beams shaped by the corrugated metal structure on the output surface^12^ have been observed, followed by the analysis of leaky-wave^13^ and the design of bull’s eye antennas in the microwave frequency^14^,^15^,^16^,^17^,^18^,^19^, as has been done in the optical frequency^2^,^20^. However, in the microwave region the period of the corrugated metal structures is about one wavelength to support leaky waves, and the angle of radiation beam was determined by the empirical formula^12^,^16^,^19^ or complicated mode expansion method^13^.

In recent years, subwavelength corrugations or grooves on metals supporting spoof SPPs with a period of approximately 1/6 wavelengths have been analyzed for slow-wave phenomena^21^,^22^,^23^,^24^,^25^,^26^. However, the radiations of leaky waves by these subwavelength corrugations have not been discussed. As spoof SPPs on the 1D structure is also a kind of transverse magnetic (TM) surface wave^11^,^13^, one could treat the subwavelength corrugations on metal as a kind of metasurface, the surface impedance of metasurface has been widely discussed and utilized to generate leaky waves, especially the sinusoidally modulated metasurfaces^27^,^28^,^29^,^30^,^31^,^32^. Conversely, the periodic structures satisfying the radiation condition could also be used to convert a propagating wave to surface wave^33^ due to the reversibility of light.

Here we apply the concept of surface impedance to the subwavelength corrugated metal structure, which originally supports the spoof SPPs, and modulate the surface impedance using the cosine function and triangle-wave function, respectively, so that the modulated structures could radiate propagating waves as a periodic leaky-wave antenna. The antenna is fed at center of the structure through a slit, connecting to a feeding waveguide, as illustrated in Fig. 1. The energy is coupled from the slit to the subwavelength corrugated structure on the output surface of the metal to propagate spoof SPPs and radiate a narrow broadside beam with high directivity. The design process is analytical, and the experimental results agree well with the numerical simulations.

## Results

### Theory and Design

Like SPPs in the optical frequency, spoof SPPs is a kind of TM surface wave. Subwavelength corrugated metal structures were proposed and investigated to support spoof SPPs, where the dispersion relation was also widely discussed^10^,^11^,^13^,^21^,^34^. By treating the subwavlength corrugated metal structure as a metasurface, we could calculate the surface impedance of different unit cells easily by analyzing the dispersion diagrams of spoof SPPs. The corrugated metal structure composed of subwavelength unit cells with the same size is quasi-uniform^35^, and support slow surface waves only^21^,^22^,^23^. Following the concept of periodic leaky-wave antenna^35^, we could then modulate the surface impedance of the corrugated structure periodically to radiate propagating waves from the spoof SPPs. Here we choose two typical periodic functions, the cosine function and the triangle-wave function, to modulate the surface impedance in order to observe the broadside radiation of leaky waves near the frequency of 17 GHz.

The distribution of surface impedance corresponding to the subwavelength corrugated metal surface modulated with cosine function is written as


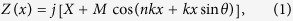


where *Z* is the surface impedance along the *x* direction that will be inductive for TM surface wave, *X* is the average surface reactance, *M* is the modulation depth, *k* is the free-space wave number at 17 GHz, 
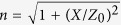
 represents the effective refractive index of surface wave, 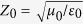 is the intrinsic impedance in free space, and *θ* is the polar angle of the radiation direction. The determination of the values for *X* and *M* will be discussed in the Methods.

Here, we set *θ* = 0 to observe a broadside radiation of leaky waves. The period of equation (1) is *p* = 2*π*/(*nk*), which is consistent with the theory of leaky waves for the first spatial harmonic:


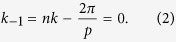


For comparison, a triangle-wave function of surface impedance is also chosen, which is written in one period as:


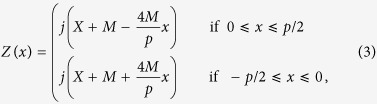


in which *X*, *M* and period *p* = 2*π*/(*nk*) are the same as those in equation (1).

**Figure 1:**
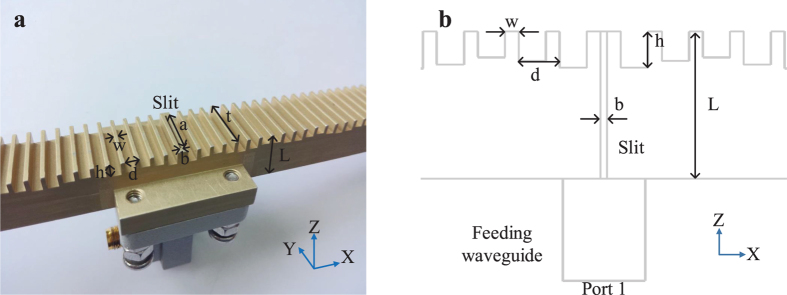
The schematic of subwavelength corrugated metal structure as a 1D periodic leaky-wave antenna. The center of the structure is a narrow slit which is connected to a feeding waveguide. The geometry parameters of the structure are given as: *w* = 1 mm, *d* = 3 mm, *h* = 2 ~ 2.9 mm, *t* = 15 mm, *L* = 11.5 mm, *a* = 12.5 mm, *b* = 0.5 mm, and *t* = 15 mm. The waveguide has a size of 13 mm × 6 mm in the cross section and a longitudinal length of 8 mm. The number of corrugations in each side from the slit is 70. (**a**) Perspective view of the sample of corrugated metal structure. (**b**) Front view of the corrugated metal structure in simulation.

A detailed structure of the antenna is illustrated in Fig. 1, which is composed of a slit surrounded by metallic corrugations on the output side, and a feeding waveguide on the input side. The slit has a size of *a* × *b* in the cross section and a longitudinal length *L*. The energy is fed from port 1 of the connected waveguide in simulation as shown in Fig. 1(b), which is then tunneled into the slit. In experiments the port 1 was replaced by an impedance-matched waveguide-to-coax adapter to feed the energy as shown in Fig. 1(a). On the output side of the slit, the energy is coupled from the slit to the corrugated conductor which supports spoof SPPs or TM surface waves. Since the surface impedance of the corrugated conductor is modulated periodically with periodic functions mentioned above, a broadside radiation of leaky waves could be observed.

The surface impedance distribution of the samples is described in above equation (1) and (3), and the relationship between depth *h* and surface impedance *Z* is described in the Methods.

The minimum reflection coefficient S11 at the designed frequency *f* is determined by the Fabry-Perot resonance of TE_10_ mode in the slit, leading to the optimal length of the slit, *L* = 11.5 mm, as shown in Fig. 1. The optimum process is described as follows. The dispersion relation of the TE_10_ mode in the slit is given as 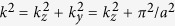, while the effective length *L*′ of the Fabry-Perot resonance is a small modification of *L* with *L*′ = *L* + Δ*L*, which has to satisfy *k*_*z*_*L*′ = *π* in order to realize the tunnel effect. Substituting *k*_*z*_*L*′ = *π* into the dispersion relation, we have


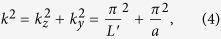


or


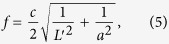


where *c* is the speed of light in vacuum. After that, for the designed frequency *f*, we could calculate the effective resonant length *L*′ as:


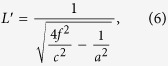


Hence, if the designed frequency is 17 GHz, the effective resonant length *L*′ is about 12.5 mm, which is then set as the initial value for the optimum process. The length of slit is finally determined as *L* = 11.5 mm, with a modification of Δ*L* = 1 mm.

### Simulations and Experiments

**Figure 2:**
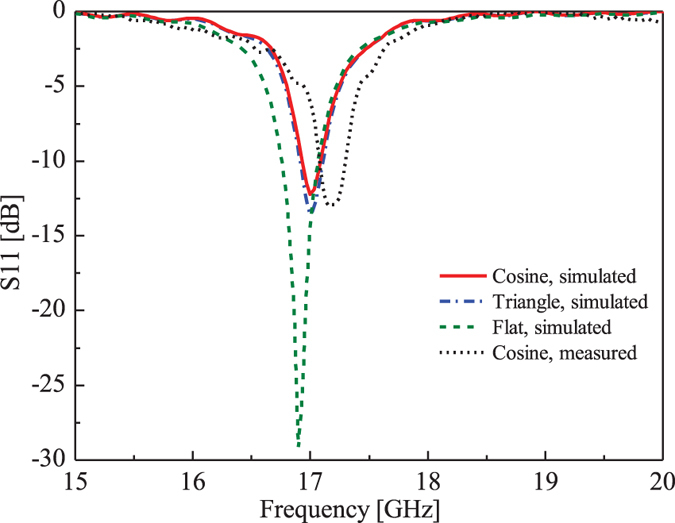
The comparison of reflection coefficients (S11).

The simulated and measured reflection coefficients (S11) are shown in Fig. 2. The red solid curve corresponds to the simulation result of the cosine distribution for the corrugated structure, and the blue dashed-doted curve refers to the simulation result of triangle-wave function distribution for the corrugated structure. For comparison, the green dashed curve gives the simulation result of a flat metal structure without corrugation. The measured result of the corrugated structure with the cosine distribution is demonstrated as the black doted line. From Fig. 2, we clearly observe that both distributions in the cosine function and triangle-wave function have a minimum reflection coefficient (S11) under −10 dB near the designed frequency 17 GHz. For the flat structure, the minimum S11 has a small shift to the low frequency and is under −25 dB. The deviation of frequency is attributed to a slightly different modification of Δ*L* as mentioned above. The smaller S11 could be explained as below. The slit could be viewed as a transmission line, and different structures surrounding the outside of slit could be treated as different load impedances. After the impedance transformation of transmission line, the input impedance on the inside of slit is better matched. For the measured reflection coefficient of the cosine distribution, there is also a small frequency shift of 200 MHz, which might be caused from the differences between the fabrication, measurement and simulation.

Spoof SPP could also be excited by monopole but the couple efficiency is relatively low^22^, a broadband of high transmission effciency could be achieved through coplanar waveguide or microstrip^24^. However, the leaky-wave radiations at broadside are not supported by above all due to the open-stopband^35^ except using slits or holes in the center^7^,^8^,^36^. Here the bandwidth of high transmission efficiency is only about 200 MHz, which is enough because the broadside radiation is only valid in a narrow band around 17 GHz due to beam scanning with frequencies^35^. The conversion from spoof SPPs to leaky-wave radiations at broadside will be discussed in the following simulations and experiments, first with far-field radiation patterns, then followed by near-field analysis.

In antenna theory concepts, the far-field radiation pattern is a graphical representation of the radiation properties of the antenna. The 2D far-field radiation patterns are often classified into E-plane and H-plane. The E-plane is defined as the plane containing the electric-field vector and the direction of maximum radiation, and the H-plane as the plane containing the magnetic-field vector and the direction of maximum radiation^37^. As the coordinates illustrated in Fig. 1, the electric -field vector of TM surface waves has only *x* and *z* components, while the magnetic-field vector has only *y* components. The maximum radiation at broadside is expected along *z* direction. So the corresponding far-field E-plane radiation pattern is in *x*-*z* plane, and H-plane is *y*-*z* plane for the structure. A view from spherical coordinate system would be more convenient, where E-plane is *ϕ* = 0°, and H-plane is *ϕ* = 90°, both *θ* varies from −180° to 180°.

**Figure 3:**
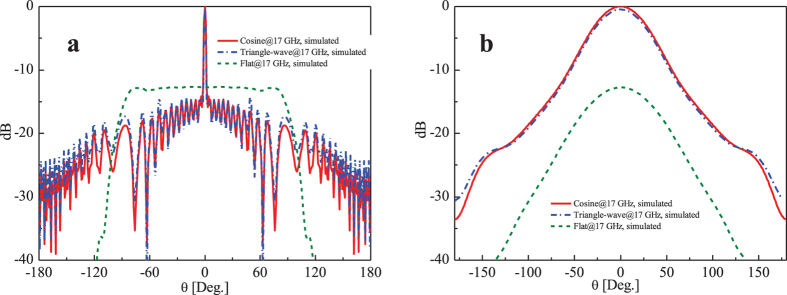
The simulated normalized radiation patterns of the subwavelength corrugated metal structure and the flat conductor. (**a**) The E-plane radiation patterns. *ϕ* = 0°. (**b**) The H-plane radiation patterns. *ϕ* = 90°.

The normalized E-plane radiation patterns of the subwavelength corrugated metal structure and flat conductor at the desired frequency 17 GHz by simulations are illustrated in Fig. 3(a), from which we observe a broadside radiation in the cases of corrugated structures. Similar to Fig. 2, the red solid curve corresponds to the simulation result of the cosine distribution for corrugated structure, and the blue dashed-doted curve refers to the result of the triangle-wave function distribution. In both cases, the narrow −3 dB beam width is about 2.2° and the side lobe levels (SLLs) are lower than −15 dB, which demonstrates there is almost same between these two type periodic functions in the design, both for the radiation pattern and S11, hence we only fabricate the cosine case for verification in the following measurements. For comparison, the green dashed curve gives the simulation result of the flat metal structure without corrugation. It could be observed that the maximum radiation enhancement for the corrugated structures in the E-plane is about 12.52 dB compared to the flat metal structure, which means the subwavelength corrugated metal structure shaped the beams from the slit to broadside radiation^12^,^13^,^14^,^15^,^16^,^17^,^18^,^38^. The simulated gain of the corrugated structure at 17 GHz is about 17.6 dB, while the simulated gain of the flat structure is about 5.08 dB. The normalized radiation patterns in the H-plane are also presented, as shown in Fig. 3(b).

**Figure 4:**
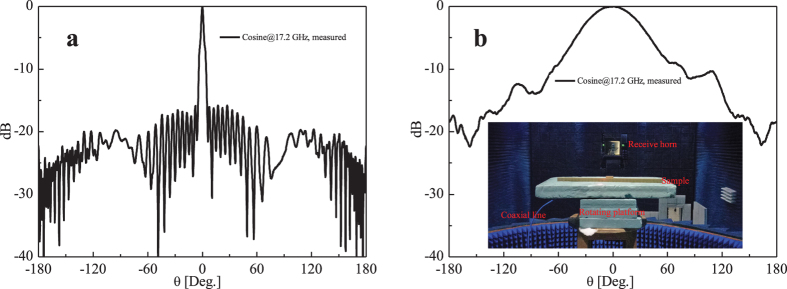
The measured normalized far-field radiation patterns of the subwavelength corrugated metal structure. (**a**) The E-plane radiation pattern. *ϕ* = 0°. (**b**) The H-plane radiation pattern. *ϕ* = 90°.

The measured radiation patterns of the cosine distribution for corrugated structure at 17.2 GHz are illustrated in Fig. 4. Figure 4(a) shows the normalized E-plane radiation pattern, from which we observe a narrow −3 dB beam width of 3°, and a SLL lower than −15 dB. The measured gain at 17.2 GHz is 16.67 dB, which is also close to the numerical results of 17.6 dB. Figure 4(b) shows the normalized H-plane radiation pattern correspondingly. The measurement process in the anechoic chamber is illustrated under the curve of the Fig. 4(b), where the sample as transmit antenna was connected with coaxial line and put on a horizontal rotating platform, and the receive horn placed in the far-field distance was at the same elevation. To measure the E-plan radiation pattern, the *x*-*z* plane of the sample illustrated in Fig. 1 was put along horizontal plane. Similarily, to measure the H-plane radiation pattern, the *x*-axis of the sample was put vertically. By rotating the platform horizontally with *θ* ranging from −180° to 180°, the corresponding radiation patterns are then obtained.

Another method of periodic leaky-wave on broadside radiation was designed with bull’s eye structure^12^,^16^,^19^. One of these structures having a period *d* near wavelength was studied at 16.5 GHz^19^, where the grooved structure has an aperture of 80 *mm* × 320 *mm*. The parameters compared with which listed in Fig. 1 are *t* = 80 *mm*, *L* = 12 *mm*, *a* = 11.54 *mm*, *b* = 2 *mm*, *w* = 14 *mm*, *d* = 16 *mm*, while *h* = 3 *mm* is a constant through the whole structure. A gain of 15.4 dB was obtained and which is 8.9 dB better than a flat structure, the SLL is about −10 dB in E-plane. Comparing our subwavelength grooved metal structure to the mentioned bull’s eye structure, we could obtain a higher gain of 17.6 dB, a better improvement of 12.52 dB than the flat structure, with lower SLL under −15 dB, narrower −3 dB beam width at broadside and smaller aperture of 30 *mm* × 420 *mm*. For further comparison, we simulated the bull’ eye structure at 17 GHz with the same aperture in commercial software, CST Microwave Studio, the geometry parameters are the same as our subwavelength structure except *h* = 3 *mm*, *w* = 13 *mm* and *d* = 15 *mm*, and the gain is about 14.5 dB at 17 GHz, with SLL of −11.6 dB and beam width of 10.9°, again the advantage was not apparent compared with our subwavelength structure. The reason for this may be that spoof SPPs have tighter spatial confinement and higher local field intensity^10^,^11^,^21^,^22^,^23^,^24^,^25^,^26^. Moreover, we could calculate surface impedances accurately to design the structure instead of optimizing by empirical formulas.

**Figure 5:**
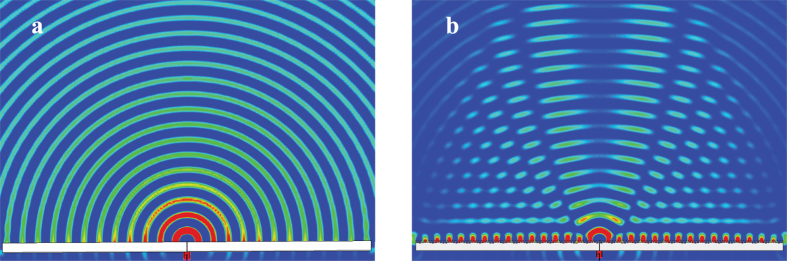
View from *x*-*z* plane for the distribution of H-field in the near field. (**a**) The flat metal structure fed by a slit. (**b**) The subwavelength corrugated metal structure fed by a slit.

The directive radiation can also be observed in the near field of leaky-waves. Figure 5(a) shows the flat structure fed with the central slit. As no subwavelength corrugated metal structure are included on the output side of the slit, the wave propagation behaves like a line source along *y*-axis. On the contrary, when the slit is surrounded by subwavelength corrugations on each side as illustrated in Fig. 5(b), more propagating waves is toward broadside. This phenomena could be understood as previous section descirbes, the energy from the slit is coupled to form spoof SPPs along the subwavelength corrugatted structures, and then the spoof SPPs are modulated periodically to radiate. The waves in the near-field are not toward broadside so obviously, the reason is that the broadside radiation is interferenced slightly from the source slit especially near the center. However, we could see that direction of waves near the structure on each side is right forward. The superposition of the two beams on each side of the center forms the symmetrical broadside beam in the far-field. A more detailed background of this phenomena could be found in previous works, where this center-fed structure was classified into periodic bidirectional antennas^35^,^36^.

**Figure 6:**
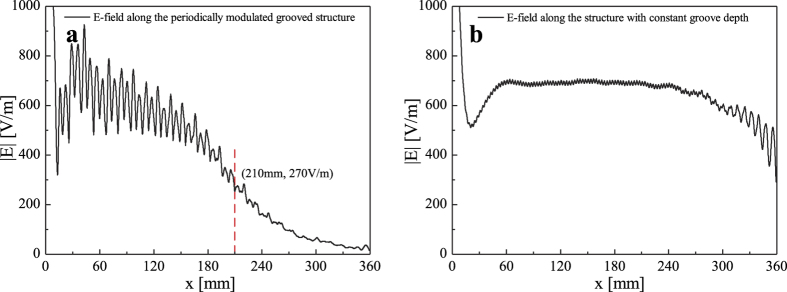
The E-field distribution along *x*-axis from *x* = 0 *mm* to *x* = 360 *mm*, with a height of 3 mm above the upsurface of structure. (**a**) The E-field distribution of the periodically modulated grooved structure. (**b**) The E-field distribution along the structure with constant groove depth, and *h* = 2 *mm* for each grooves.

The gain can be improved by increasing the number of corrugations in each side from the slit. Here we choose the number as 70, the length of the unit cell is 3 mm, hence the span of the structure is about 70 × 3 × 2, i.e., 420 mm. The choice of the span is determined by two reasons. First, 70 corrugations in each side is enough to obtain the side lobe leve under −10 dB for the far-field radiation pattern from simulations. Second, the amplitude of E-field along x-axis is declined rapidly after about 70 corrugations. Figure 6(a) shows the distribution of E-fields along the x-axis from the center to the end of the periodically modulated structure with about 120 corrugations or length of 360 mm, from which we observe the amplitude of E-field reduces by almost a factor of 1/e at *x* = 210 *mm*, with about 270 V/m. Hence, choosing 210 mm or 70 corrugations is enough for the design. Figure 6(b) demonstrates the distribution of the E-fields along the x-axis from the center to the end of the uniform spoof SPPs structure with h = 2 mm for each grooves. Here a longer propagation distance for the spoof SPPs is observed. In both Fig. 6(a,b), the steep parts of the curves in the beginning are caused by the center-fed and coupling from the slit to grooved structure. After all, the choice of 70 corrugations is then determined from the observation of the decay in the near field and the desired SLL under −10 dB.

## Discussion

Conventionally, subwavelength corrugated metal structures have been studied for supporting spoof SPPs, but here we studied the radiation of leaky waves using this structure. By treating the subwavelength corrugated conductor as metasurface to support TM surface waves and calculating the surface impedance of different unit cells, we then modulated the surface impedance periodically as periodic leaky wave antennas to radiate leaky waves to the desired directions. A broadside radiation is achieved near the designed frequency (17 GHz). It should be mentioned that this design is not limited to the broadside radiation, and the width *t* could be adjusted freely since it is insensitive to the dispersion relations^23^,^24^. The proposed method could also be applied to metal disk to design a high gain bull’s eye antenna^18^,^38^ or observe phenomena of pseudo Bessel beams^32^ both in gigahertz and terahertz frequencies.

## Methods

**Figure 7:**
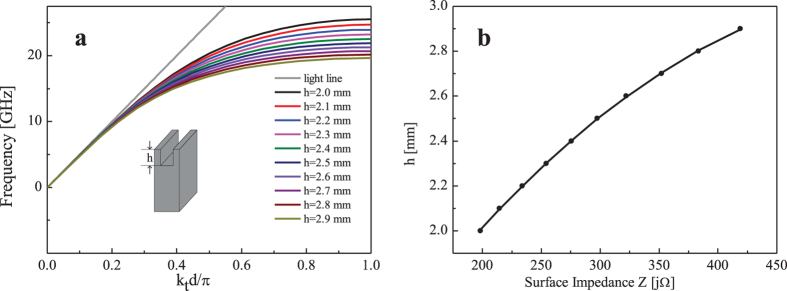
The dispersion relations of the unit cells for different *h* and the fitted curve for the surface impedance. (**a**) Dispersion relations of the unit cells. (**b**) Surface impedances of different depths *h* at 17 GHz.

Spoof SPPs propagating on the subwavelength corrugated metal structures are TM surface wave, so that we can calculate the surface impedances of the corrugated conductors with different depths from the dispersion diagrams. By simulating the unit cells of corrugated conductor with depths *h* = 2 ~ 2.9 mm using the Eigenmode Solver in commercial software, CST Microwave Studio, we could get the dispersion diagrams of different unit cells, as shown in Fig. 7(a). Then, the range of the surface impedance (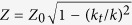) of all unit cells at 17 GHz, determines parameters *X* = 308.552, *M* = 110.38, *n* = 1.292 in equation (1). Meanwhile the datas at 17 GHz in Fig. 7(a) is curve fitted to a function of *h* = −7.634 × 10^−6^ × |*Z*|^2^ + 0.008753 × |*Z*| + 0.569444 as illustrated in Fig. 7(b), which can be used to design the depth distribution of the subwavelength corrugated metal structure combining with equation (1).

## Additional Information

**How to cite this article**: Cai, B. G. *et al.* Leaky-Wave Radiations by Modulating Surface Impedance on Subwavelength Corrugated Metal Structures. *Sci. Rep.* **6**, 23974; doi: 10.1038/srep23974 (2016).
